# Impact of HMGB1/TLR Ligand Complexes on HIV-1 Replication: Possible Role for Flagellin during HIV-1 Infection

**DOI:** 10.1155/2012/263836

**Published:** 2012-06-06

**Authors:** Piotr Nowak, Samir Abdurahman, Annica Lindkvist, Marius Troseid, Anders Sönnerborg

**Affiliations:** ^1^Department of Infectious Diseases, Institution of Medicine, Karolinska University Hospital and Karolinska Institutet, 14186 Stockholm, Sweden; ^2^Department of Clinical Microbiology, Institution of Laboratory Medicine, Karolinska University Hospital and Karolinska Institutet, 14186 Stockholm, Sweden; ^3^Department of Infectious Diseases, Oslo University Hospital, Ullevål, 0424 Oslo, Norway

## Abstract

*Objective*. We hypothesized that HMGB1 in complex with bacterial components, such as flagellin, CpG-ODN, and LPS, promotes HIV-1 replication. Furthermore, we studied the levels of antiflagellin antibodies during HIV-1-infection. *Methods*. Chronically HIV-1-infected U1 cells were stimulated with necrotic extract/recombinant HMGB1 in complex with TLR ligands or alone. HIV-1 replication was estimated by p24 antigen in culture supernatants 48–72 hours after stimulation. The presence of systemic anti-flagellin IgG was determined in 51 HIV-1-infected patients and 19 controls by immunoblotting or in-house ELISA. *Results*. Flagellin, LPS, and CpG-ODN induced stronger HIV-1 replication when incubated together with necrotic extract or recombinant HMGB1 than activation by any of the compounds alone. Moreover, the stimulatory effect of necrotic extract was inhibited by depletion of HMGB1. Elevated levels of anti-flagellin antibodies were present in plasma from HIV-1-infected patients and significantly decreased during 2 years of antiretroviral therapy. *Conclusions*. Our findings implicate a possible role of HGMB1-bacterial complexes, as a consequence of microbial translocation and cell necrosis, for immune activation in HIV-1 pathogenesis. We propose that flagellin is an important microbial product, that modulates viral replication and induces adaptive immune responses *in vivo*.

## 1. Introduction

Antiretroviral therapy (ART) suppresses efficiently the replication of human immunodeficiency virus type 1 (HIV-1) to undetectable levels with standard techniques in most treated patients, but there is still an ongoing low-grade replication in most or all patients [1]. Also, immune activation is a central feature of progressive HIV-1 infection [2–4], and although the degree of immune activation is decreased during ART, it is not normalized [5]. The pathogenic mechanisms for the persistent immune activation remain further to be determined. This is especially important since studies suggest that the remaining immune activation may cause organ damage, for example, an increased risk for cardiovascular diseases and possibly neurocognitive dysfunction [5, 6].

The gastrointestinal (GI) immune system seems to play a central role in the pathogenesis of immune activation [7]. The early dramatic depletion of CD4+ T cells from the gut mucosa may drive immune activation, as this mucosal immune damage impairs the normal barrier function and allows increased translocation of bacterial products from the gut lumen into the circulation [8]. We and others have shown that microbial translocation is present in HIV-1 infection through increased plasma LPS levels in subjects with progressive disease and that the levels are decreased by ART [9–11].

Furthermore, we and others have implied that the alarmin high-mobility group binding 1 protein (HMGB1) modulates HIV-1 replication *in vitro* and contributes to the activation of immune system [12–14]. Thus, plasma HMGB1 levels are elevated in HIV-1-infected patients and reduced with effective ART [11, 15]. HMGB1 is released from damaged or necrotic cells to the extracellular milieu, in which it may act as a potent proinflammatory marker by stimulating cytokine expression in monocytes and endothelial cells [16, 17]. HMGB1 per se does not seem to have a pro-inflammatory activity [18, 19] but has a high affinity to form complexes with other molecules such as LPS and CpG-DNA [20]. These complexes are likely to bind to various receptors, including TLR4 and TLR9, and promote a large variety of inflammatory and immunological responses [20, 21].

The aim of our study was to explore whether complexes of HMGB1 and TLR ligands, such as flagellin, could synergistically induce HIV-1 replication in a promonocytic cell line.

## 2. Methods

### 2.1. Ethic Statement

All research involving human participants has been conducted according to the principles expressed in the Declaration of Helsinki. Patients gave their informed written consent and the study protocol was approved by the Regional Ethics Committee in Stockholm, Sweden (Dnr 2005/3:10).

### 2.2. Reagents

Lipopolysaccharide (LPS) and (phorbol-12-myristate-13-acetate) PMA were obtained from Sigma (St. Louise, MO, USA), IL-1*β* from R&D Systems (Minneapolis, MN, USA), and CpG-ODN type B (ODN2006), purified flagellin (*S.typhimurium*), and anti-flagellin (FliC) antibodies from InvivoGen (San Diego, CA, USA and Abcam, Cambridge, UK). Recombinant HMGB1 (HM-116) was purchased from HMGBiotech (Milan, Italy) or from R&D systems (Minneapolis, MN, USA). We also used recombinant HMGB1 [19] that was a kind gift from Professor Helena Erlandsson-Harris CMM/KI, Stockholm.

### 2.3. Cell Cultures

U1 cells, a subclone derived from U937 cells, were obtained through the AIDS Research and Reference Reagent Program (NIAID, NIH). The U1 cells are chronically infected with HIV-1 and are characterized by low constitutive levels of virus expression that can be upregulated by several cytokines and phorbol esters. The cells were maintained in RPMI medium (Gibco) supplemented with 10% fetal calf serum, glutamine, and antibiotics. Cells were seeded at 200 000 cells/mL in 96-well plates and complexes/TLR-ligands/controls were added and incubated for 48 or 72 hours.

### 2.4. Patients

Patients (*n* = 51) given ART, followed at the Department of Infectious Diseases, Karolinska University Hospital, Stockholm, and 19 healthy controls were included. Patients' recruitment was based on sample availability as well as virologic response after 2 years of ART. Thirty-three individuals had undetectable viral load and 18 had detectable viraemia (nonresponders) after 2 years of treatment. This cohort (42 of 51 patients) has been described previously [11]. The age and sex distribution of the patients and controls was similar (median age 38 years, 52% women).

### 2.5. Preparation of Necrotic Cell Extracts

Necrotic extracts were obtained as previously described [22]. Briefly, necrosis was induced in peripheral blood mononuclear cells (PBMCs) from healthy donors (30 × 10^6^ cells/mL) by exposing the cells to six cycles of freezing and thawing. Cell debris was removed by centrifugation and the supernatant was passed through a 0.2 *μ*m membrane and collected. The concentration of HMGB1 in necrotic extracts was 40 *μ*g/mL, as estimated by immunoblot (data not shown). Furthermore, 250 *μ*L of necrotic extract was incubated with polyclonal anti-HMGB-1 antibodies (Abs) from ABCAM (Cambridge, UK). Samples were incubated at 4°C for 16 hours. As control, nonspecific Abs (Rabbit polyclonal IgG) was utilized. Immune complexes were removed by adding 25 *μ*L of Sepharose A/G to the extract, incubated for 1.5 hours at 4°C, and centrifuged. The supernatant was collected and the procedure was repeated again with 25 *μ*L sepharose for 1 hour at 4°C.

### 2.6. Preparation of HMGB1 Complexes

**Figure 1:**
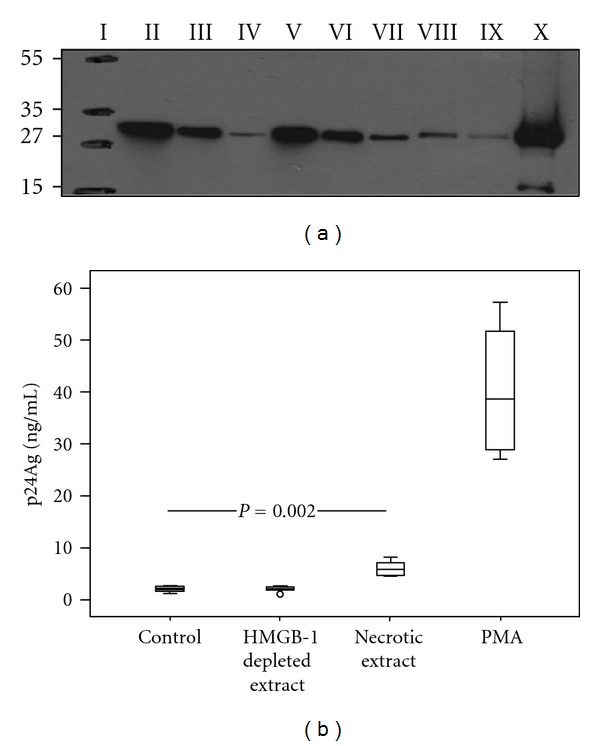
HMGB1 present in necrotic extract induces HIV-1 replication in U1 cells. (a) Western blot of cell supernatants (necrotic extracts) obtained after freeze-thawing cycles of peripheral blood mononuclear cells (PBMC) (30 × 10^6^ cells/mL) from healthy donors: Molecular weight marker (I); supernatants after immune depletion of HMGB1 with nonspecific rabbit polyclonal antibody (II); depletion with anti-HMGB1 antibody −5 *μ*g (III) and 10 *μ*g (IV); necrotic extract loaded 20 *μ*L (V), 10 *μ*L (VI), and 5 *μ*L (VII); 100 ng (VIII) and 75 ng (IX) of recombinant HMGB1; cell debris (X). Numbers to the left depict positions of molecular mass markers (in kDa). (b) Levels of HIV p24 protein in cell culture supernatants after 72 h incubation of U1 cells with necrotic extract (HMGB1 concentration 1 *μ*g/mL): HMGB1-depleted necrotic extract and mock cells. PMA served as a positive control (20 nM). The levels of viral replication were approximately 2-fold higher after stimulation by necrotic extract compared to the mock cells (*P* = 0.002). Results from three independent experiments in duplicates are presented.

**Figure 2:**
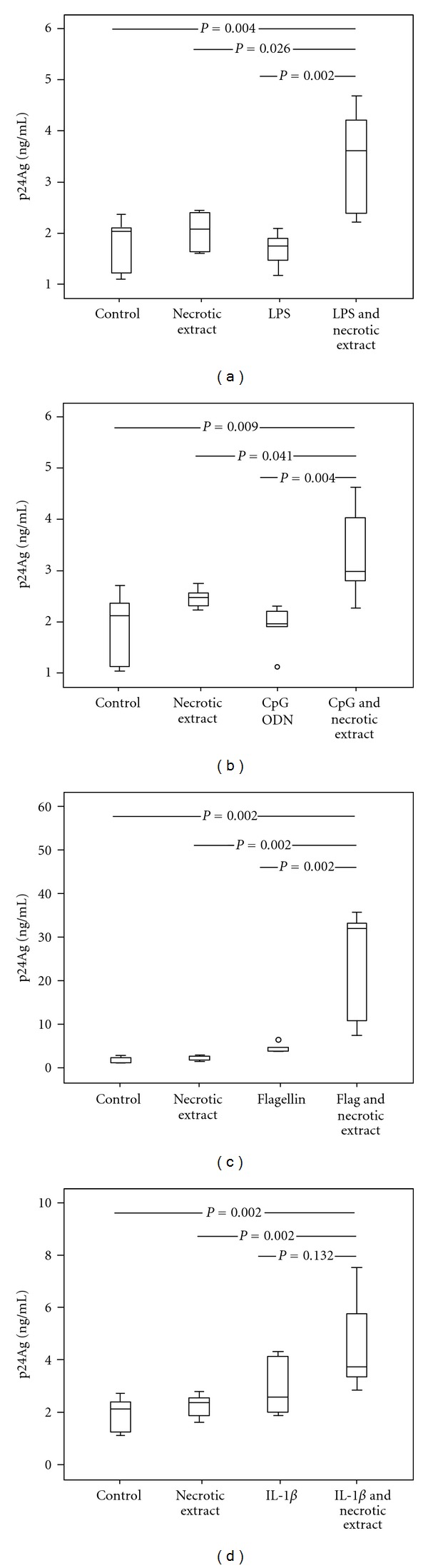
Necrotic extract and TLR-ligands in complexes upregulate viral replication in U1 cells. U1 cell cultures were stimulated with necrotic extract (HMGB1 concentration 1 *μ*g/mL) and Toll-like receptor ligands: LPS 10 ng/mL (a), CpG-ODN 1 *μ*g/mL (b), flagellin 50 ng/mL (c), and IL-1*β* 0.25 ug/mL (d) alone or in complexes. Supernatants from mock cells served as controls. Supernatants were collected from cell cultures after 72 hours. Results from three independent experiments in duplicates are presented.

**Figure 3:**
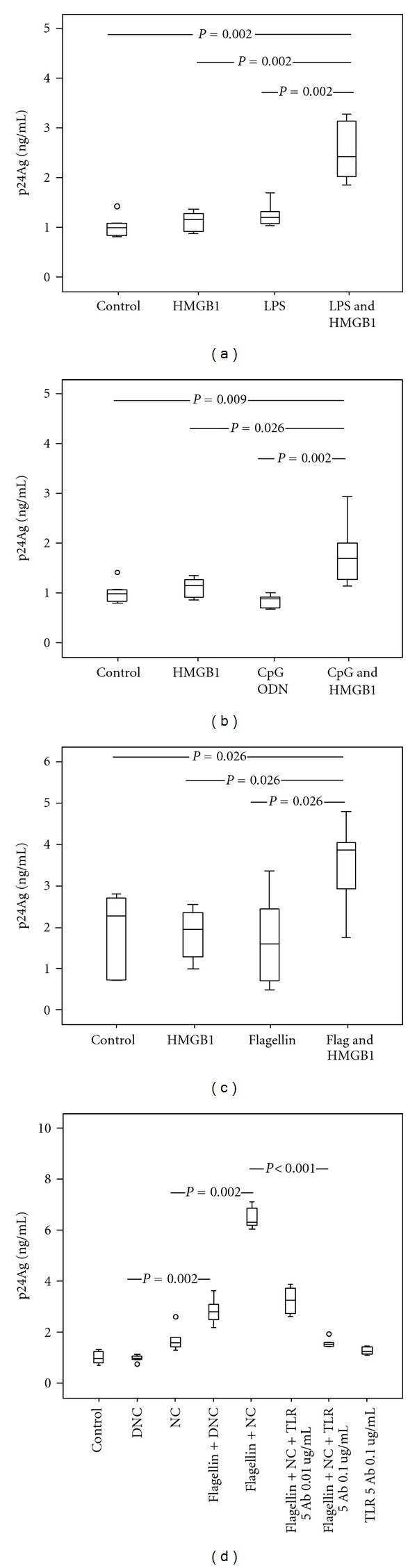
Interacting effect of recombinant HMGB1 and TLR-ligand complexes in U1 cells. Inhibition of flagellin complexes induced HIV-1 replication by anti-TLR5 antibodies. U1 cells were stimulated with recombinant HMGB1 (1 *μ*g/mL) and TLR ligands: LPS 10 ng/mL (a), CpG-ODN 1 *μ*g/mL (b) and flagellin 10 ng/mL (c) alone or in complexes. (d) U1 cells were incubated with 0.1 and 0.01 ug/mL anti-TLR5 antibodies (TLR5 Ab) for 1 hour and then exposed to necrotic extract (NC) and HMGB1-depleted necrotic extract (DNC), alone or in complexes with flagellin (10 ng/mL). HIV-1 replication was estimated after 48 hours of incubation. A dose-dependent inhibition of flagellin-necrotic extract complexes stimulatory effect is present in wells pretreated with anti-TLR5 antibodies (*P* for trend < 0.001). Results from three independent experiments in duplicates are shown.

Necrotic extract or HMGB1was mixed with the TLR-ligands, LPS, CpG-ODN, IL-1*β*, and flagellin in PBS in different concentrations and incubated for 16 hours at 4°C. Concentrations are given in Figures 1–3. The suboptimal stimulatory concentrations (capable to trigger HIV replication from U1 cells) of necrotic extract as well as LPS, flagellin, CpG-ODN, and Il-1*β* were estimated in a series of experiments (data not included). Complexes were also mixed and denatured by heating at 95°C for five minutes to verify the stimulatory effect of complex formation on U1 cells.

### 2.7. Characterization of HMGB1 with Immunoblotting

Equal volumes of necrotic cell extracts, HMGB1-depleted necrotic cell extracts, as well as recombinant HMGB1 proteins were resolved on 10–20% Tris/glycine gel and transferred onto nitrocellulose membrane (Invitrogen, Carlsbad, USA). The membranes were then incubated overnight with anti-HMGB1 Abs at 1 : 2000 dilution. The following day, the membranes were incubated 1 h with horseradish-peroxidase (HRP-) conjugated secondary antibody (GE, Healthcare), raised against rabbit IgG at 1 : 10,000 dilution. The proteins were finally visualized using ECL reagents (GE, Healthcare).

### 2.8. Immunoblotting of Antiflagellin Antibodies

Approximately 1.88 *μ*g of recombinant flagellin was twofold serially diluted (4 series) and subjected to gel electrophoresis on 10–20% precasted SDS-PAGE gel (Invitrogen) in Tris/glycine/SDS buffer. Similarly, bacterial extracts of flagellated *E. coli *strain O126:H2 and aflagellate *E. coli *O21:H-(CCUG catalog number 11425 and 11326, kind gift from Professor Andrej Weintraub, Clinical Microbiology/KI, Stockholm) that were prepared freshly from an overnight inoculation were also resolved on SDS-PAGE gel as described previously. The proteins were then electroblotted onto iBlot gel transfer stacks nitrocellulose membranes, using the iBlot dry blotting system (Invitrogen), as recommended by the manufacturer. After blocking the nitrocellulose membranes for 1 hour in blocking buffer (PBS supplemented with 0.05% Tween and containing 10% nonfat milk), the blots were probed with primary antibodies overnight at 4°C with a slow agitation. As primary antibody, serum from HIV-1-infected or control subjects diluted 1 : 1000, monoclonal, or polyclonal anti-flagellin Abs was used. The following day, the membranes were washed with PBS containing 0.05% (vol/vol) Tween and bound antibodies were then detected by using HRP-conjugated secondary Abs (Pierce) against human IgG in 1 : 10,000 dilution. Protein bands were visualized by chemiluminescence (Thermo Scientific). To confirm the protein bands, two immunoblotted membranes from HIV-1-infected patients and control subjects were stripped off and reprobed with mouse monoclonal antibody directed against flagellin. Bound antibodies were then detected by using an HRP-conjugated secondary antibody, raised against mouse (DAKO; 1 : 4.000).

### 2.9. Specific and Total Antibodies Measurement

Antibody titers against flagellin, measles, and total IgG levels were assessed by ELISA. An in-house anti-flagellin-specific IgG ELISA was developed using purified flagellin monomers from *S. typhimurium* (InvivoGen). It has been previously shown that human sera have a similar recognition pattern of flagellin monomers whether isolated from flagellated *E. coli *or *S. typhimurium* [23]. Briefly, microwell plates (MWP) were coated overnight with purified flagellin from *S. typhimurium* (25 ng/well). The following day, plasma samples from HIV-1-infected and control subjects diluted 1 : 1000 were applied to wells coated with flagellin. After incubation and washing, the MWPs were incubated with HRP-conjuggated anti-human IgG. For total IgG ELISA, the manufacturer's procedure was followed (MABTECH, Nacka, Sweden). The Enzygnost Measeles virus IgG ELISA kit (Behring, Germany) was utilized for quantification of antimeasles antibodies.

### 2.10. Plasma HIV-1 RNA Quantification and CD4+/CD8+ T-Cell Counts

Plasma HIV-1 RNA levels (COBAS Amplicor test Roche Molecular Systems; USA; detection limit 40 copies/mL) and T-cell counts (flow cytometry) were evaluated as part of clinical routine.

### 2.11. HIV-1 Replication Assay

Supernatants were collected at indicated time points and tested for the presence of HIV p24 antigen with Architect i2000 HIV-1 Ag/Ab combo detection system (Abbott Diagnostics, Abbott Park, IL, USA). The p24 concentration was calculated based on the several standard dilutions of p24 protein included in each run.

### 2.12. Statistics

Data are presented as median, interquartile range, and total range. Differences between groups were analysed with the Mann-Whitney *U*-test, and intragroup changes from baseline to the end of the study were evaluated by Wilcoxon test. Jonckheere-Terpstra test was used for trend analyses and correlation analyses were performed using the Spearman method. A two-tailed significance level of 0.05 was used. The statistical analyses were performed with SPSS software, version 15.0 (SPSS Inc, Chicago, USA).

## 3. Results

### 3.1. Necrotic Cellular Extract Upregulates HIV-1 Replication in U1 Cells

To determine the impact of an endogenous signal, associated with cell injury, on HIV-1 replication, we generated soluble necrotic extracts from healthy donors PBMCs. Western blot confirmed the presence of large amounts of HMGB1 in the extracts. Additionally HMGB1-depleted extracts were obtained by immune depletion utilizing specific anti-HMGB1 antibodies (Figure 1(a)). In the initial experiment, the U1 cells were exposed to necrotic extract, HMGB1-depleted extract, and PMA, respectively. The HIV p24 antigen concentration in the cell supernatants was measured after 72 hours (Figure 1(b)). The levels of viral replication were approximately 2-fold higher after stimulation by necrotic extract compared to the mock cells (*P* = 0.002). The stimulation with PMA gave a 10-fold higher viral replication than stimulation with necrotic extract. Notably, addition of necrotic extract depleted of HMGB1 did not result in an increase of viral replication, as compared to the controls, suggesting that HMGB1 crucially contributes to the stimulatory effect of the necrotic extract.

### 3.2. Interacting Effect of TLR Ligands and Necrotic Extract on HIV-1 Replication in U1 Cells

Thereafter, we stimulated the U1 cells with necrotic extract, TLR ligands (LPS, flagellin, CpG-ODN), and IL-1*β* alone or with the complexes of necrotic extract and the TLR ligands or IL-1*β*. Notably, stimulation with all the TLR ligands, in combination with necrotic extract, resulted in a higher viral replication than stimulation with necrotic extract or TLR ligands alone (Figure 2). Hence, stimulation with LPS, CpG-ODN and IL-1*β* in complexes with necrotic extract resulted in a 1.5–2-fold-increased viral replication compared to each component alone, whereas flagellin in combination with necrotic extract resulted in a 7-fold increased replication compared to flagellin alone and a 13-fold-increased replication compared to necrotic extract alone. The preheating of complexes prior incubation with cells resulted in abrogation of stimulatory signal, implying that the active compound relies on intact protein structure (data not included).

### 3.3. Interacting Effect of TLR Ligands and HMGB1 on HIV-1 Replication in U1 Cells

In order to explore if HMGB1 could mimic the synergistic effects of necrotic extract-TLR ligands, we challenged the U1 cells with complexes consisting of HMGB1 and bacterial substances. Indeed, stimulation with microbial products (LPS, flagellin, or CpG-ODN) in combination with HMGB1 resulted in a higher viral replication than stimulation with HMGB1 or TLR ligands alone (Figures 3(a)–3(c)). Stimulation with LPS, flagellin, and CpG-ODN in combination with HMGB1 resulted in a 1.5–2-fold-increased viral replication compared to each component alone, although the stimulatory effect was not as prominent as with TLR ligands in combination with necrotic extract.

### 3.4. Dose-Dependent Inhibition of Flagellin by Anti-TLR5

It is known that immune response to flagellin is mediated by TLR5. To investigate whether anti-TLR5 antibodies could block the inducing effects of flagellin, we first preincubated U1 cells with anti-TLR5 antibodies and subsequently added the necrotic extract complexed with flagellin. Flagellin in combination with HMGB1-depleted extract gave a 3-fold-increased viral replication compared to depleted extract alone, whereas flagellin in combination with necrotic extract gave a 4-fold increase compared to necrotic extract alone (Figure 3(d)). Preincubation of U1 cells with anti-TLR-5 antibodies before addition of necrotic extract-flagellin complexes resulted in a dose-dependent inhibition of this stimulatory effect (*P* for trend < 0.001). Addition of anti-TLR5 antibodies alone did not affect viral replication.

### 3.5. Detection of Antiflagellin Antibodies in HIV-1-Infected Patients

**Figure 4:**
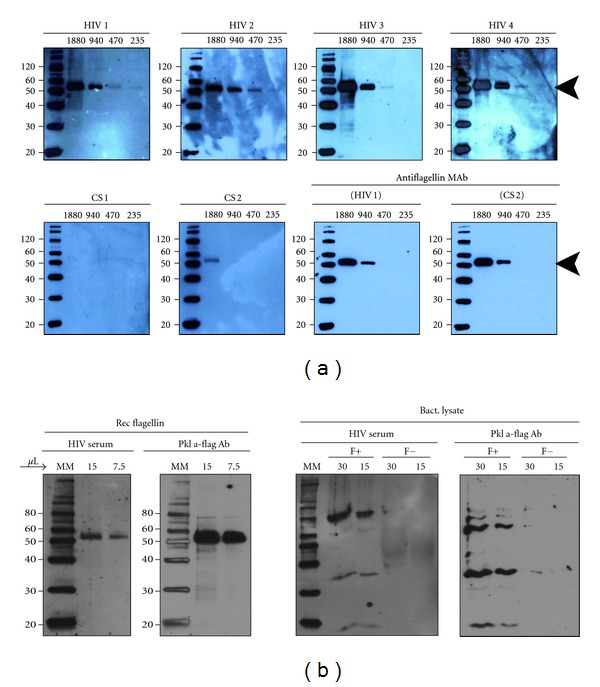
HIV-1-infected patients exhibit elevated levels of flagellin-specific antibodies. (a) Approximately 1.8 *μ*g of recombinant flagellin was twofold serially diluted (4 series) and resolved on 10–20% SDS-PAGE gel, transferred onto a nitrocellulose membrane, and detected with immunoblot assay. Serum from HIV-1-infected or control subjects diluted 1 : 1000 was used as a primary antibody. Each panel is a separate immunoblot of the same recombinant flagellin detected with serum from HIV-1 patients (HIV 1, HIV 2, HIV 3, HIV 4) or control subjects (CS 1, CS 2). To confirm equal sample loading and protein transfer, immunoblotted membranes from HIV 1 and CS 2 were stripped off and reprobed with monoclonal antibody directed against flagellin (blots in the lower panel). The experiments were performed using sera from ten HIV-1-infected patients and four control subjects. These presented data are representative for all immunoblots. The position of recombinant flagellin protein is indicated with an arrow to the right. Numbers to the left depict positions of molecular mass markers (in kDa). (b) Recombinant flagellin (column 1) and lysates of flagellated or aflagellate *E. coli* (columns 3 and 4) were subjected to SDS-PAGE and immunoblotted using serum from HIV infected patient (column 1 and 3, diluted 1 : 1000) as primary antibody or polyclonal anti-flagellin antibody (columns 2 and 4, diluted 1 : 1000). The membrane in column 1 was stripped off and reprobed with polyclonal anti-flagellin antibody (column 2). The numbers above the figures indicate the amount of sample loaded in each well (in *μ*L). MM, magic marker (Invitrogen); F+: flagellated; F−: aflagellate whole bacterial lysate; Pkl: polyclonal; Ab: antibody. Numbers to the left depict positions of molecular mass markers (in kDa).

Encouraged by the *in vitro* data we aimed to evaluate whether flagellin is a potentially important antigen *in vivo *during HIV-1 infection. Therefore, serum samples from HIV-1-infected patients and control subjects were used to measure the level of flagellin-specific antibodies by Western blot analysis. When diluted 1 : 1000, all of the sera samples from the HIV-1-infected patients analyzed exhibited easily detectable bands that recognized the first two dilutions of flagellin derived from *S. typhimurium* (Figure 4(a), upper panels). A relative increase in flagellin-specific IgG in HIV patients was observed if serum samples were diluted 1 : 500. In contrast, in only one control subject (CS#2) flagellin was detected faintly at the highest dilution (Figure 4(a), lower panels). Although semiquantitative analysis of detected bands was not performed, the levels of flagellin-specific IgG observed were in all cases strikingly elevated in HIV-1-infected patients. Similar pattern of anti-flagellin IgG was observed when plasma instead of sera was used. In order to address the specificity of the flagellin IgG, we subjected the bacterial lysates from flagellated and aflagellate *E. coli* to protein separation on the SDS-PAGE gel. The Western blotting with HIV-1 serum (as a primary antibody) showed similar pattern as when the polyclonal anti-flagellin antibody was used confirming that the specificity of the antibodies was not limited to the recombinant protein (Figure 4(b)).

**Figure 5:**
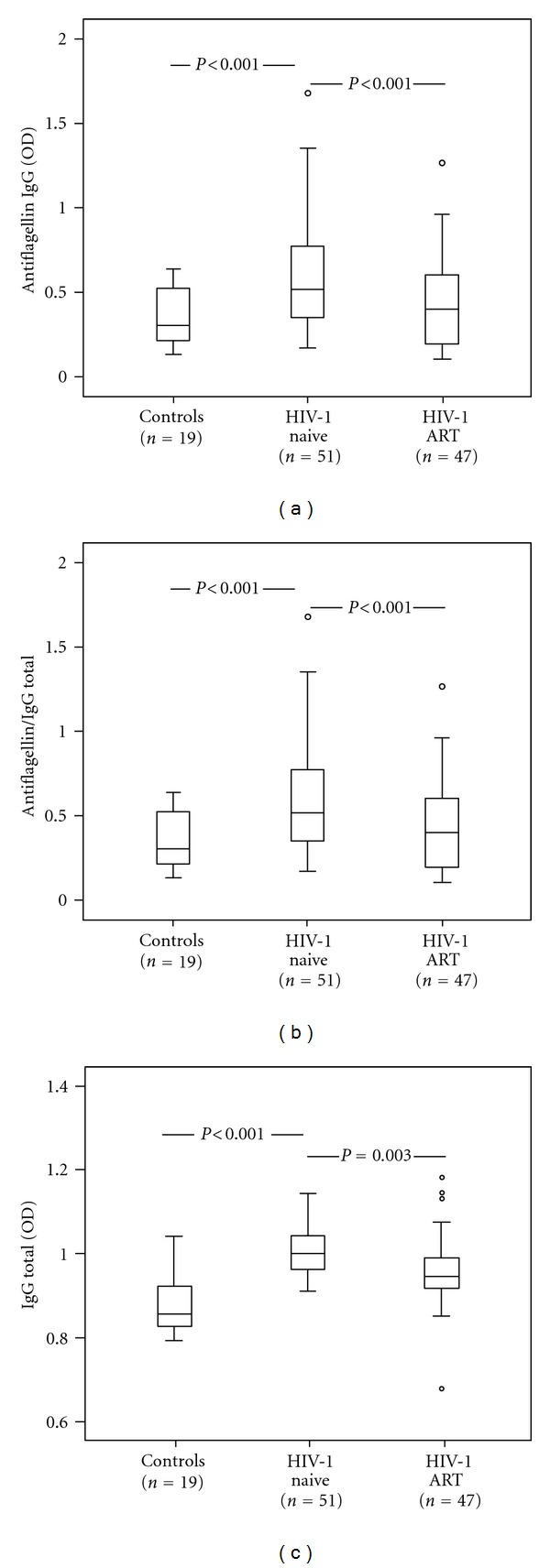
Elevated levels of anti-flagellin IgG are reduced during ART. Plasma levels (OD) of anti-flagellin IgG (a), ratio anti-flagellin IgG/total IgG (b), and total IgG (c) in healthy controls, HIV-1-infected individuals before (= naïve) and after two years of ART (antiretroviral therapy). The plasma samples were diluted 1:1000. The data are presented as median 25–75 interquartile range and total range. *P* values refer to intergroup differences.

Furthermore, we used the anti-flagellin ELISA to evaluate the levels of flagellin IgG in plasma of HIV-1-infected patients before and after two years of ART. At baseline significantly elevated levels of flagellin antibodies were found in HIV-1-infected patients as compared to controls (*P* < 0.001) (Figure 5(a)). This difference persisted (*P* < 0.001) when the flagellin antibodies were adjusted to the total IgG (Figure 5(b)), suggesting that the elevation of flagellin antibodies was not due to hypergammaglobulinemia. Moreover, analysis of antimeasles antibodies in 10 patients with severe immune deficiency (CD4+ T-cell counts ≤ 200) supported that the elevation of the flagellin antibodies was not caused by polyclonal activation Supplementary Table 1 (see supplementary material available online at doi:10.1155/2012/263836). The levels of flagellin IgG, total IgG, and the ratio flagellin IgG/total IgG were significantly reduced after two years of ART for the whole group (*P* < 0.001, *P* = 0.03, and *P* < 0.001, resp. Figures 5(a)–5(c)). Additionally a significant reduction of flagellin IgG levels was observed also when the patients were subdivided into those with successful ART and the nonresponders who had remaining low levels of viral replication two years after initiating the ART (*P* = 0.009; *P* = 0.001, resp.). The total IgG levels after ART did not decrease in nonresponders as they did in successfully treated patients (*P* < 0.001) (data not shown).

We found no correlation between the levels of flagellin IgG and the viral load nor the CD4/CD8 T-cell counts. In contrast, among the subgroup of 42 patients in whom we had earlier analysed HGMB1 and LPS in plasma, significant correlations were found between the levels of flagellin IgG and LPS (*r* = 0.32; *P* = 0.02) as well as between the flagellin IgG/total IgG ratio and LPS (*r* = 0.25; *P* = 0.007) (data not shown).

## 4. Discussion

Microbial translocation has been described in different conditions like inflammatory bowel disease, neutropenia, and chronic viral infections [6, 24]. In HIV-1 infection, the proof for the translocation of bacterial products is based mainly on LPS data [9, 11, 25, 26]. However, the original observation that increased LPS levels were associated with both activated memory CD8+ T cells and enhanced IFN-*α* levels implies the involvement of other factors [9].

We therefore hypothesized that HMGB1 could be such a link between the microbial products and hyperinflammation [27]. Mounting evidence shows that HMGB1 does not act alone but forms stable potent proinflammatory complexes with other molecules, such as bacterial products or single stranded DNA [18–20, 28]. Since we have earlier shown that HMGB1 alone activates latent HIV-1 replication *in vitro *[12], we decided to expand our analysis to the effect of HGMB1 in complexes with bacterial products. Here, we present that HMGB1 in complex with the TLR ligands (LPS, CpG-ODN, flagellin) and IL-1*β* induce viral replication in a promonocytic cell line, U1 cells. The data obtained with both the HMGB1 derived from necrotic extract as well as recombinant protein yielded similar results, although the stimulatory signals associated with necrotic HMGB1 were more potent. This is not surprising as other endogenous danger signals should be anticipated in this process [20]. The reduction of the stimulatory effect by depletion of HGMB1 also supports our hypothesis that HMGB1 is an important component of these complexes.

These *in vitro* findings brought our attention to flagellin as a potent activator of HIV-1 replication alone or in complexes with HMGB1. Bacterial flagellins are present in all motile bacteria and play an important role in mediating gut inflammation associated with infection by enteric pathogens or in inflammatory bowel diseases [29]. Their proinflammatory activity is exerted mainly through TLR5 [30, 31]. It has been recently demonstrated that flagellin is the major antigen activating innate and adaptive immune response in intestinal inflammation observed in Crohn's disease [32, 33]. Disruption of the intestinal barrier promotes translocation of flagellated commensal bacteria across the epithelium driving the activation of innate immune cells residing in the lamina propria. This phenomenon results also in abnormal exposure of immune cells to flagellin, a process that may influence the balance and function of the different T-cell subsets present in the gut-associated immune system (GALT) and promote inflammation [29, 34].

The contribution of flagellin to immune activation during HIV-1 infection has been anticipated [7], but the data are scarce. Thus, exposure of PBMCs to flagellin resulted in activation of T cells predominantly of central memory and effector memory phenotype [35]. Moreover, flagellin is able to induce HIV-1 gene expression in resting memory CD4+ T cells that are considered as a key cellular HIV-1 reservoir in infected individuals [36].

Our *in vitro* and *in vivo *findings are clearly in line with the hypothesis of an important role of flagellin in HIV-1 pathogenesis. Thus, we demonstrated that flagellin complexes are able to significantly stimulate the HIV-1 replication, at least from cells of monocytic origin. Furthermore, our finding that elevated levels of anti-flagellin IgG are present in HIV-1-infected individuals anticipates that this observation has *in vivo* implications. Hence, we show not only presence of elevated levels of flagellin antibodies before the initiation of ART but also a reduction after two years of ART suggesting decreased exposure to the antigen probably due to partial restoration of gut-blood barrier [25, 37]. The hypergammaglobulinemia present during the HIV-1 infection cannot be solely responsible for the elevation of flagellin IgG levels as the normalisation to the IgG did not influence the results.

An elevated adaptive immune response to flagellin has been previously observed in conditions associated with gut barrier dysfunction such as Crohn's disease and short bowel syndrome [23, 24] and relates to the severity of Crohn's disease [38]. Also, Kamat et al. [39] reported recently the presence of a subgroup of anti-flagellin antibodies (anti-CBir1) in 4/26 HIV-1-infected patients with CD4+ T-cells counts <300 cells/ul and high LPS levels. The CBir1 flagellin has been identified as an immune dominant flagellin in Crohn's disease and linked to Clostridia species. Interestingly a recently presented work has shown alterations in bacterial composition of microbiota during HIV-1 infection with significantly lower ratio of Clostridia taxa in faeces obtained from HIV-1-infected patients as compared to controls [40]. Although our assay is based on the recognition of *Salmonella typhimurium* flagellin which spans the two well conserved N- and C-terminal domains of flagellin [41] enabling us the detection of broader range of flagellin antibodies, additional studies are needed to determine the antibody specificity. Furthermore, our results deserve future studies of adaptive flagellin immune response in a larger cohort of HIV-1-infected individuals.

In summary, the novelty of our findings is that the bacterial products and HMGB1 form active complexes which can efficiently not only create a proinflammatory milieu but also directly trigger viral replication in infected cells. This synergistic effect may require lower levels of the interacting substances when present in complexes, as suggested by others [19]. We also report that flagellin has to be considered as a microbial product that can contribute to the immune activation during the HIV-1 infection. The formation of HMGB1/TLR ligand complexes has direct implications on immune activation, particularly in late stage of disease, where cell destruction and necrosis are dominant phenomena due to CD4+ T-cell loss, opportunistic infections, and other pathological conditions [27].

## Supplementary Material

The detailed characteristics of patients' immune and virological status in relation to the levels of anti-flagellin and anti-measles IgG.
