# Supplementary material for: Impact of HMGB1/TLR Ligand Complexes on HIV-1 Replication: Possible Role for Flagellin during HIV-1 Infection
Source: Int J Microbiol. 2012 Jun 6;2012:263836. doi: 10.1155/2012/263836 (PMC3375154; doi:10.1155/2012/263836)
Supplement: Supplementary file 1 — The detailed characteristics of patients' immune and virological status in relation to the levels of anti-flagellin and anti-measles IgG. [file 263836.f1.pptx]

## Slide 1
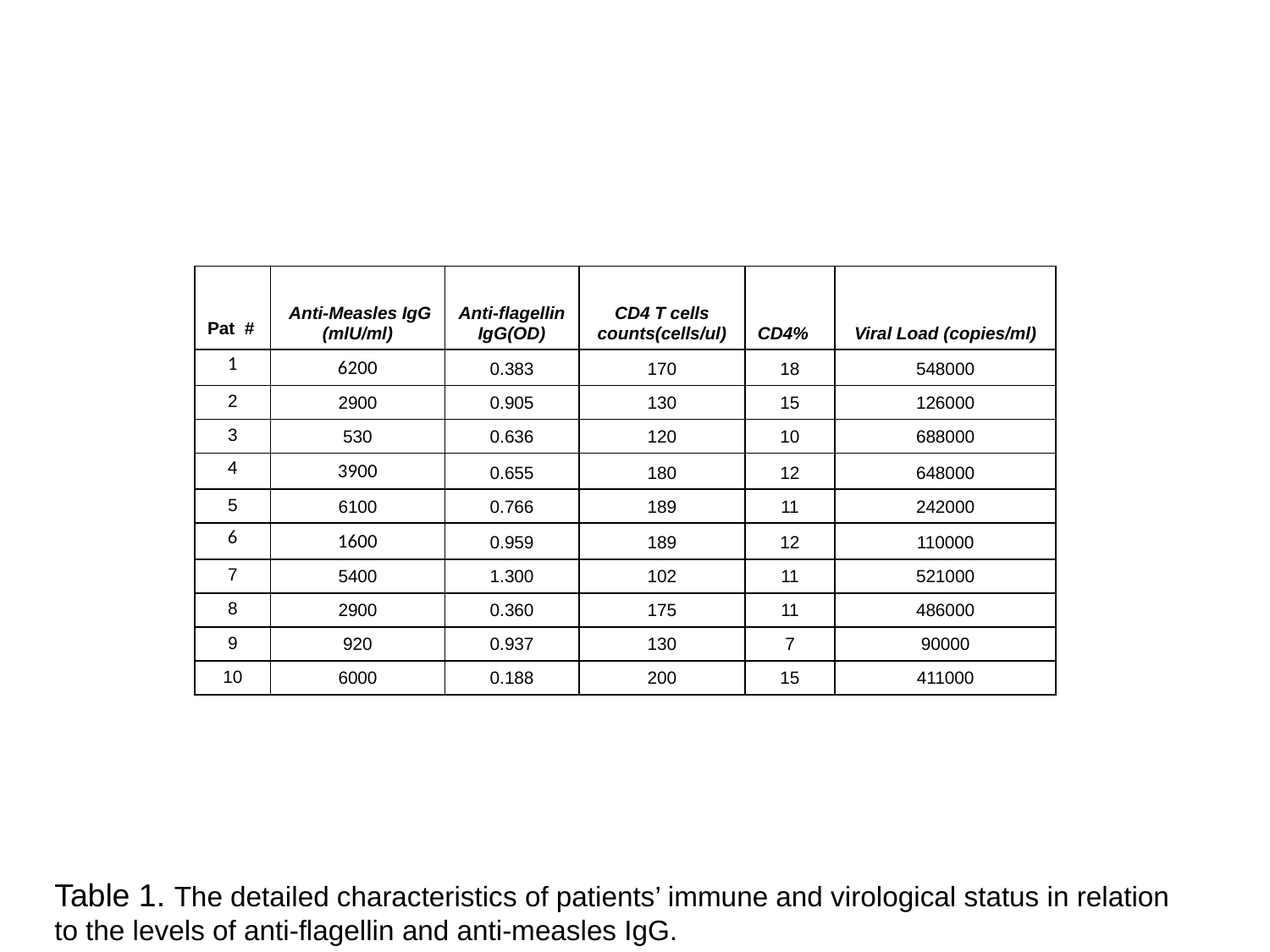

| Pat # | Anti-Measles IgG (mlU/ml) | Anti-flagellin IgG(OD) | CD4 T cells counts(cells/ul) | CD4% | Viral Load (copies/ml) |
| --- | --- | --- | --- | --- | --- |
| 1 | 6200 | 0.383 | 170 | 18 | 548000 |
| 2 | 2900 | 0.905 | 130 | 15 | 126000 |
| 3 | 530 | 0.636 | 120 | 10 | 688000 |
| 4 | 3900 | 0.655 | 180 | 12 | 648000 |
| 5 | 6100 | 0.766 | 189 | 11 | 242000 |
| 6 | 1600 | 0.959 | 189 | 12 | 110000 |
| 7 | 5400 | 1.300 | 102 | 11 | 521000 |
| 8 | 2900 | 0.360 | 175 | 11 | 486000 |
| 9 | 920 | 0.937 | 130 | 7 | 90000 |
| 10 | 6000 | 0.188 | 200 | 15 | 411000 |
Table 1. The detailed characteristics of patients’ immune and virological status in relation 	to the levels of anti-flagellin and anti-measles IgG.
